# Supplementary material for: TPH1 and 5-HT7 Receptor Overexpression Leading to Gemcitabine-Resistance Requires Non-Canonical Permissive Action of EZH2 in Pancreatic Ductal Adenocarcinoma
Source: Cancers (Basel). 2021 Oct 22;13(21):5305. doi: 10.3390/cancers13215305 (PMC8582390; doi:10.3390/cancers13215305)
Supplement: Supplementary file 1 [file cancers-13-05305-s001.zip › cancers-1400509-supplementary.pdf]

Supplementary Materials: TPH1 and 5-HT7 Receptor Overexpression Leading to Gemcitabine-Resistance Requires Non-Canonical Permissive Action of EZH2 in Pancreatic Ductal Adenocarcinoma

Prakash Chaudhary, Diwakar Guragain, Jae-Hoon Chang and Jung-Ae Kim

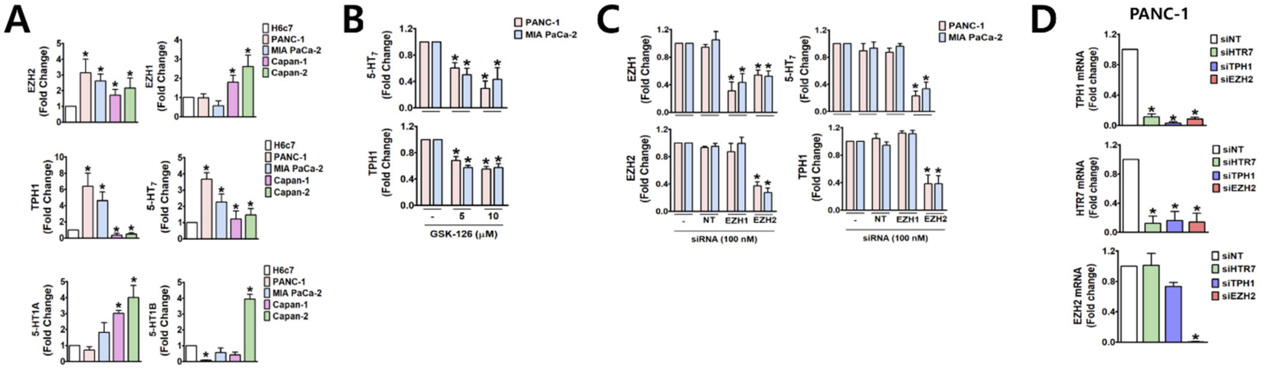

Figure S1. Effect of HTR7, TPH1, or EZH2 KD on the expression of protein or mRNA of EZH2 and 5-HT system. (**A**) Basal protein levels of EZH1, EZH2, TPH1, and 5-HT receptor subtypes (5-HT7, 5-HT1A, 5-HT1B) from three independent experiments were quantitated in H6c7, PANC-1, MIA PaCa-2, Capan-1 and Capan-2 cells. (**B**) and (**C**) After PANC-1 and MIA PaCa-2 cells were treated with GSK-126 for 48 h (**B**) or transfected with EZH1 and EZH2 siRNAs (**C**)., the expression levels of TPH1 and 5-HT7 were quantitated. (**D**) Effects of siRNA transfection specific to HTR7, TPH1, or EZH2 on mRNA expression of TPH1, HTR7 and EZH2 in PANC-1 cells. All the immunoblots were performed in three independent experiments, and relative density of each protein was quantitated. Data points in bar graphs are the means ± S.E.M. * $p < 0.05$ compared to vehicle-treated control.

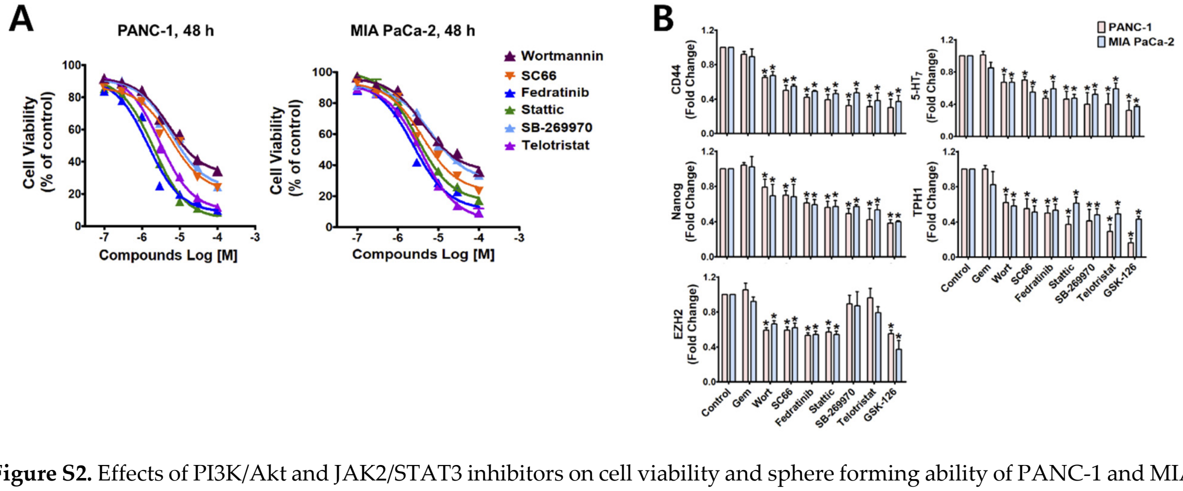

Figure S2. Effects of PI3K/Akt and JAK2/STAT3 inhibitors on cell viability and sphere forming ability of PANC-1 and MIA PaCa-2 cells. (**A**) Cell viability was measured in PANC-1 and MIA PaCa-2 cells that were treated with 7 different concentrations of stattic, fedratinib, wortmannin, telotristat, or SB-269970 for 48 h. (**B**) Quantitation of relative densities of immunoblots (5-HT7, TPH1, CD44, Nanog and EZH2) in PANC-1 and MIA PaCa-2 spheres treated with inhibitors (1 μM) of EZH2-TPH1-5-HT7 axis and related signaling molecules. * $p < 0.05$ compared to the vehicle-treated control.

Fig 1B

Lane 1: H6c7
Lane 2: PANC-1
Lane 3: MIA PaCa-2
Lane 4: Capan-1
Lane 5: Capan-2

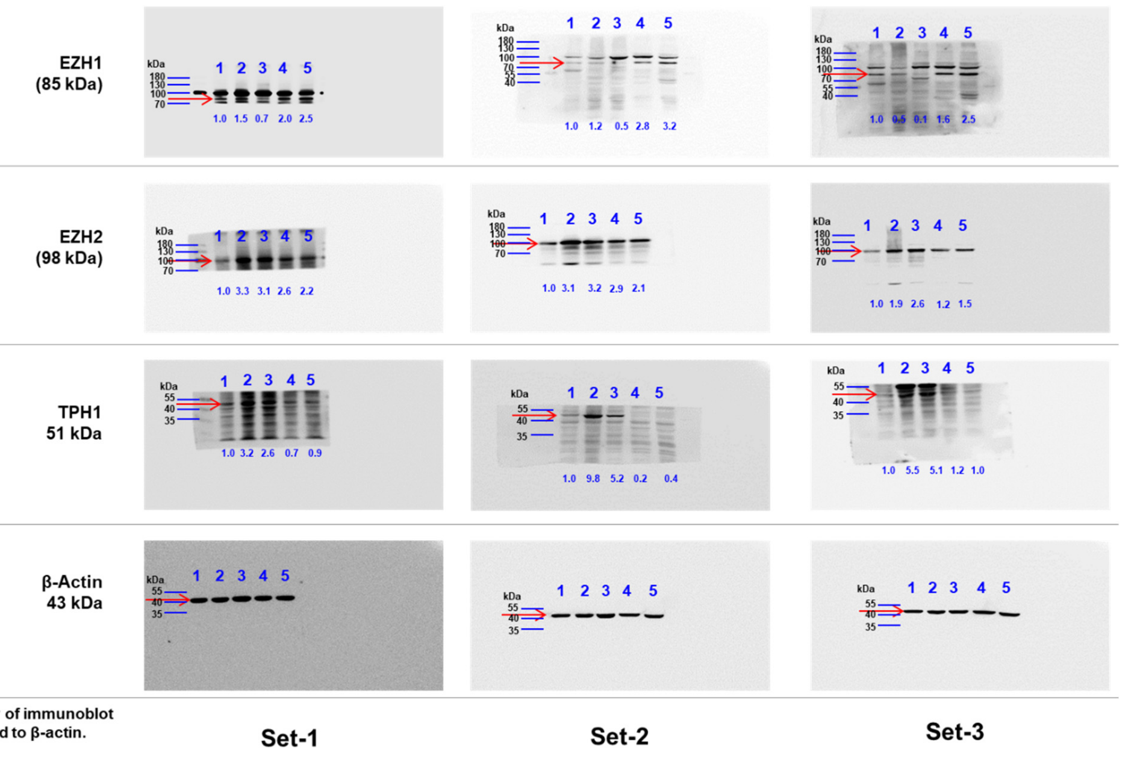

Each band intensity of immunoblot has been normalized to β-actin.

Fig 1B continued

Lane 1: H6c7
Lane 2: PANC-1
Lane 3: MIA PaCa-2
Lane 4: Capan-1
Lane 5: Capan-2

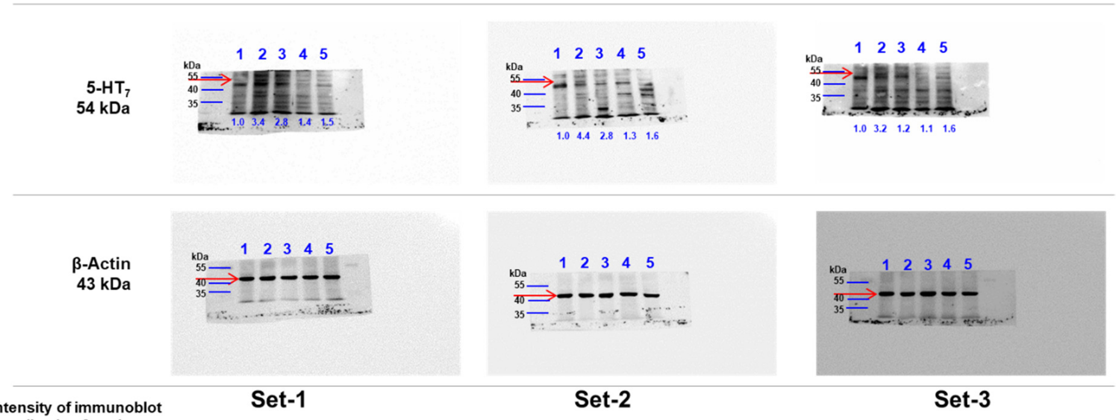

Each band intensity of immunoblot has been normalized to β-actin.

Fig 1B continued

Lane 1: H6c7
Lane 2: PANC-1
Lane 3: MIA PaCa-2
Lane 4: Capan-1
Lane 5: Capan-2

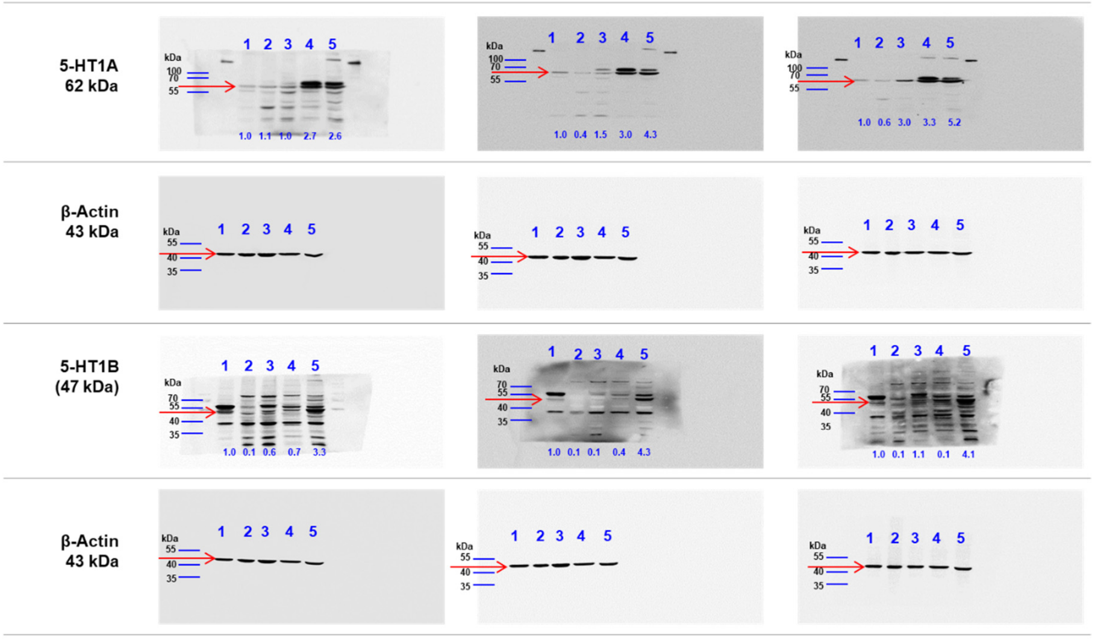

Each band intensity of immunoblot has been normalized to β-actin.

Set-1 **Set-2** **Set-3**

Fig 1C

Lane 1: Vehicle
Lane 2: DZNep (5 μM)
Lane 3: DZNep (10 μM)
Lane 4: GSK126 (5 μM)
Lane 5: GSK126 (10 μM)

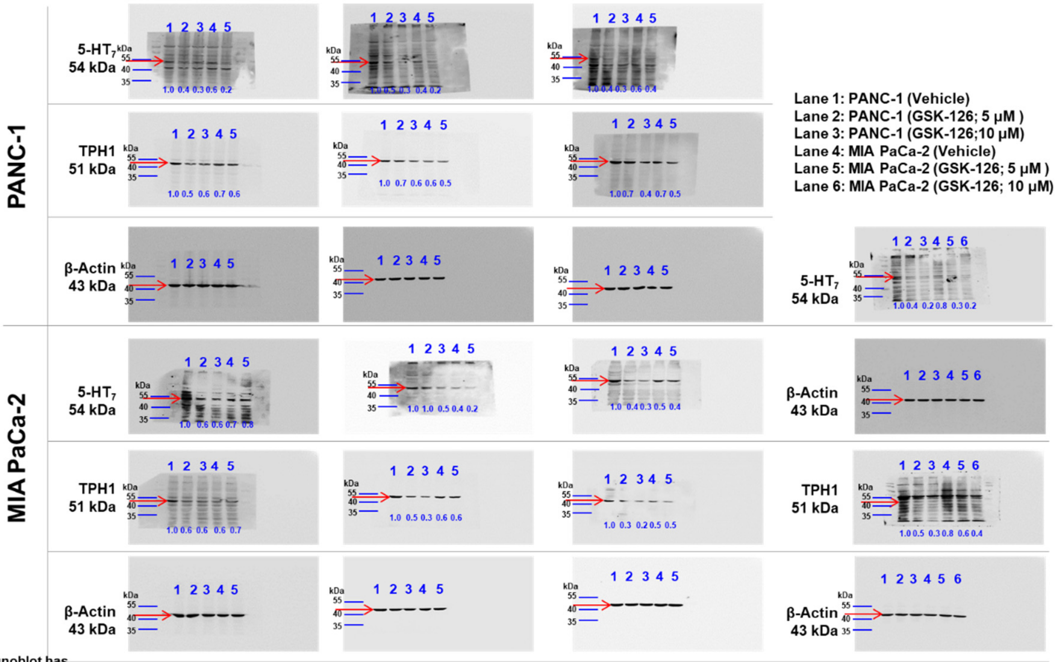

Each band intensity of immunoblot has been normalized to β-actin.

Set-1 **Set-2** **Set-3** **Set-4**

Fig 1 D

Lane 1:Mock
Lane 2:siNT
Lane 3:siHTR7
Lane 4:siTPH1
Lane 5:siEZH2

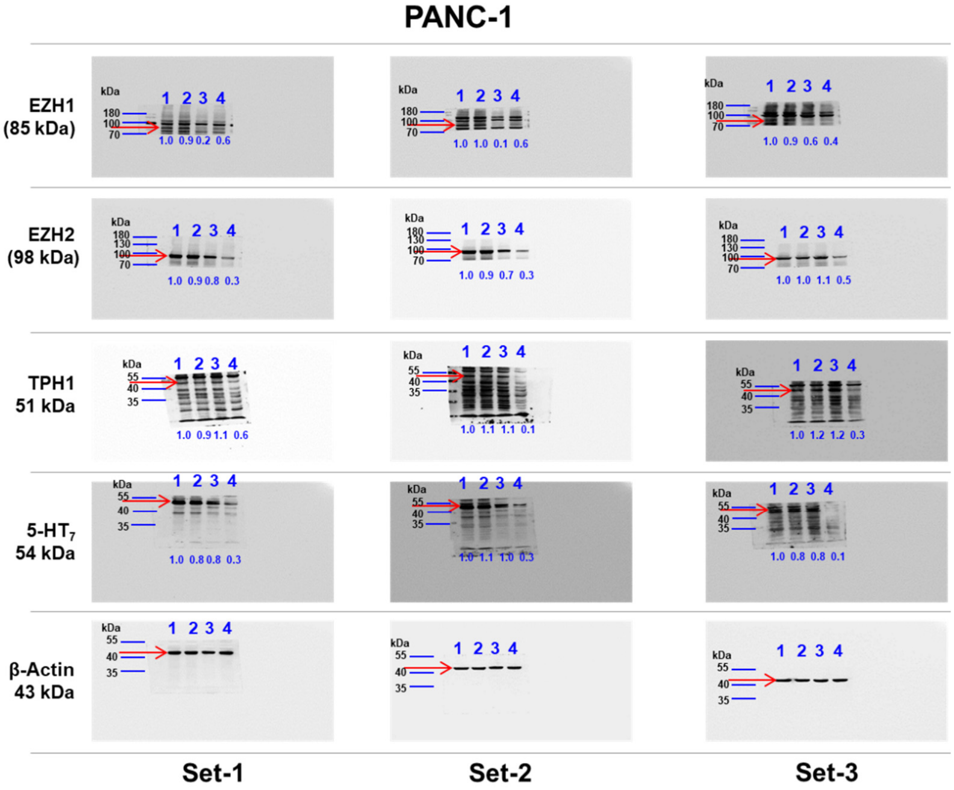

Each band intensity of immunoblot has been normalized to β-actin.

Fig 1 D Continued

Lane 1: Mock
Lane 2: siNT
Lane 3: siHTR7
Lane 4: siTPH1
Lane 5: siEZH2

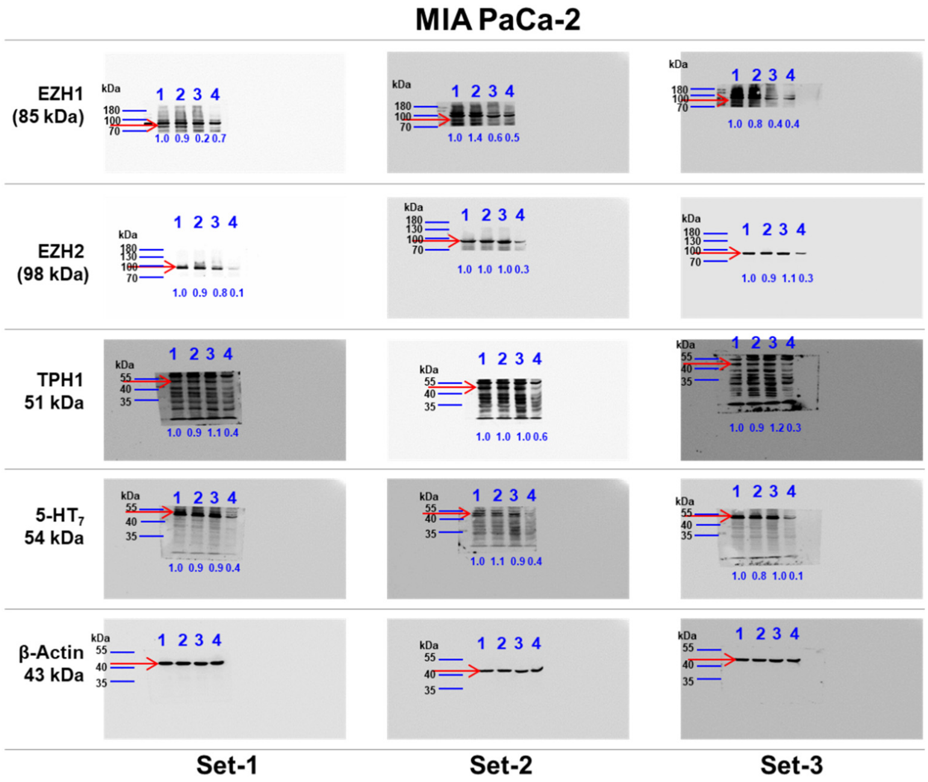

Each band intensity of immunoblot has been normalized to β-actin.

Fig 1 E

Lane 1: siNT
Lane 2: siHTR7
Lane 3: siTPH1
Lane 4: siEZH2
Lane 5: siNT
Lane 6: siHTR7
Lane 7: siTPH1
Lane 8: siEZH2

Lane 1-4 (PANC-1)
Lane 5-8 (MIA PaCa-2)

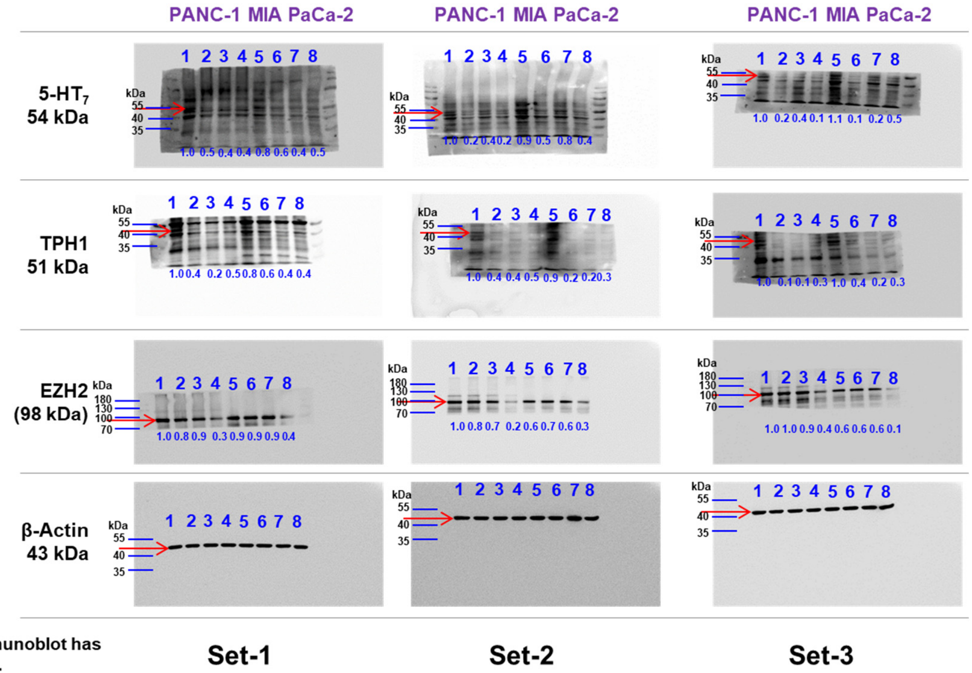

Each band intensity of immunoblot has been normalized to β-actin.

Fig 1 H

Lane 1: 5-HT (10 μM), 0 h
Lane 2: 5-HT (10 μM), 24 h
Lane 3: 5-HT (10 μM), 48 h
Lane 4: 5-HT (10 μM), 72 h
Lane 5: 5-HT (10 μM), 96 h
Lane 6: 5-HT (10 μM), 120 h
Lane 7: 5-HT (10 μM), 144 h

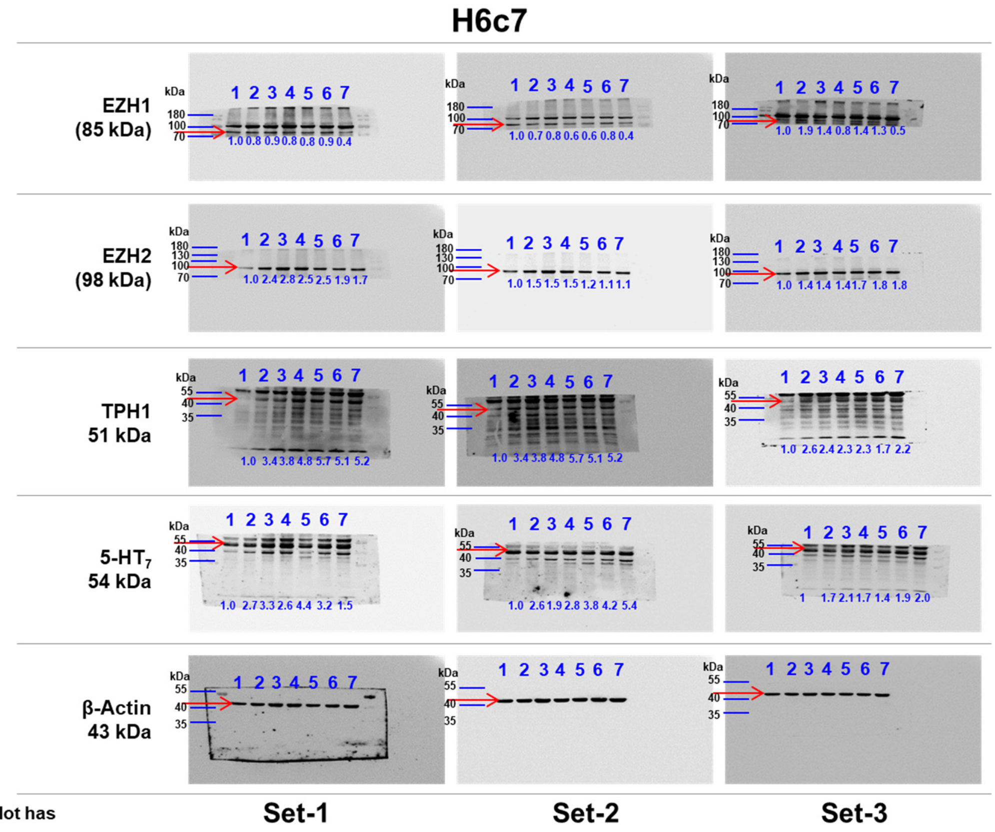

Each band intensity of immunoblot has been normalized to β-actin.

Fig 1 H continued

Lane 1: 5-HT (10 µM), 0 h
Lane 2: 5-HT (10 µM), 24 h
Lane 3: 5-HT (10 µM), 48 h
Lane 4: 5-HT (10 µM), 72 h
Lane 5: 5-HT (10 µM), 96 h
Lane 6: 5-HT (10 µM), 120 h
Lane 7: 5-HT (10 µM), 144 h

Each band intensity of immunoblot has been normalized to β-actin.

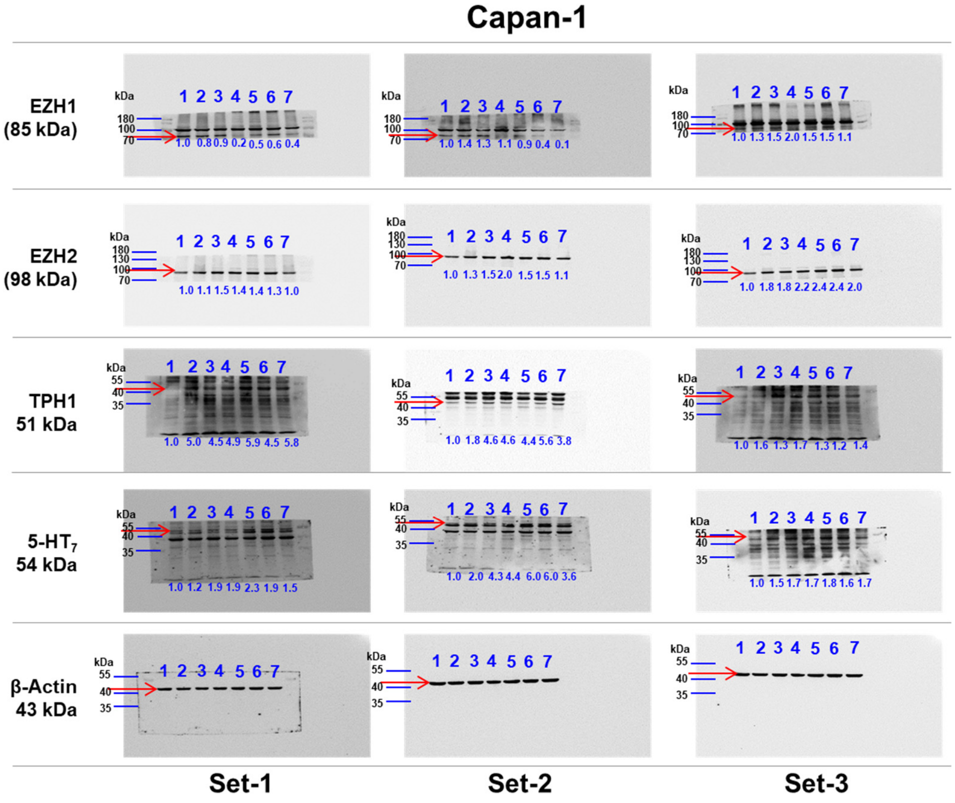

Figure S3. Uncropped Western Blot images for Figure 1.

Fig 3 A

PANC-1

Lane 1: Mock
Lane 2: siNT
Lane 3: siHTR7
Lane 4: siTPH1
Lane 5: siEZH2

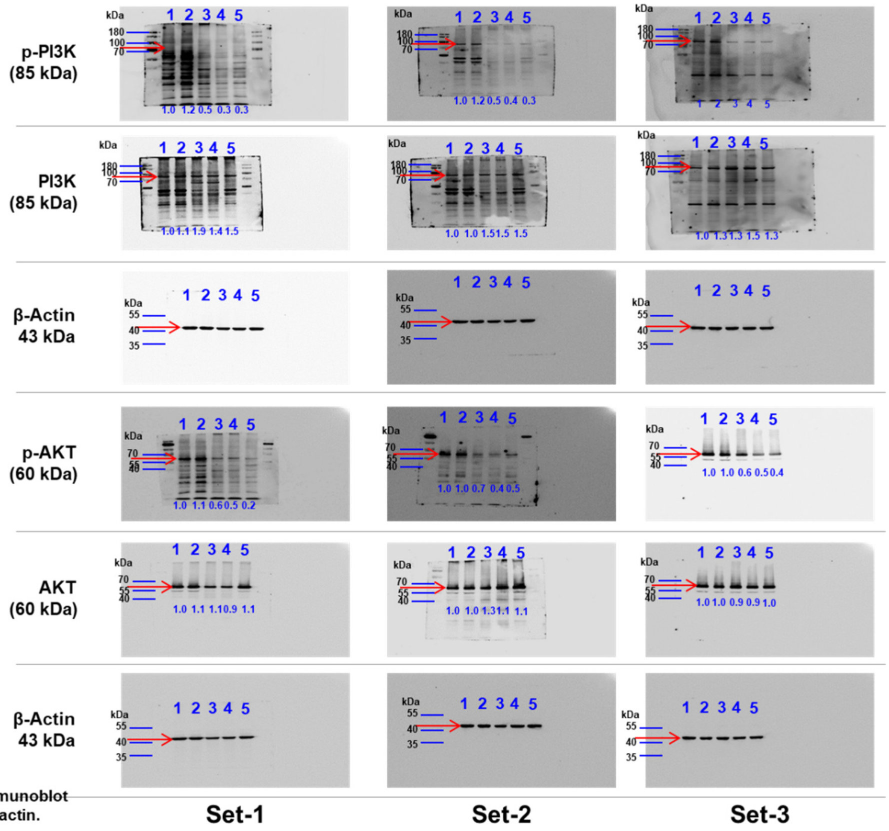

Each band intensity of immunoblot has been normalized to β-actin.

Fig 3 A continued

PANC-1

Lane 1: Mock
Lane 2: siNT
Lane 3: siHTR7
Lane 4: siTPH1
Lane 5: siEZH2

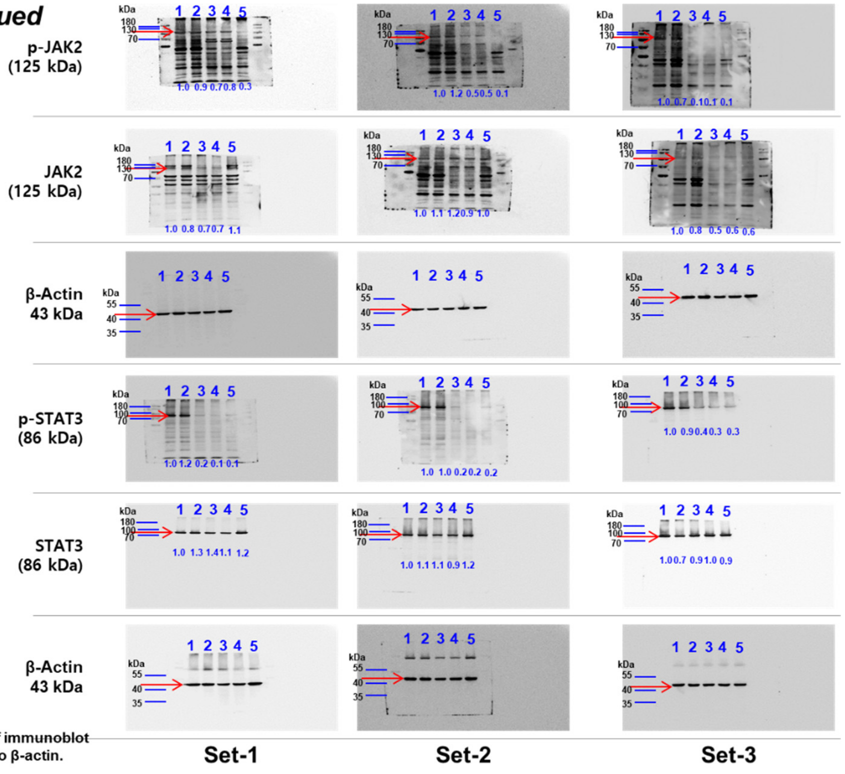

Each band intensity of immunoblot has been normalized to β-actin.

Fig 3 A continued

MIA PaCa-2

Lane 1: Mock
Lane 2: siNT
Lane 3: siHTR7
Lane 4: siTPH1
Lane 5: siEZH2

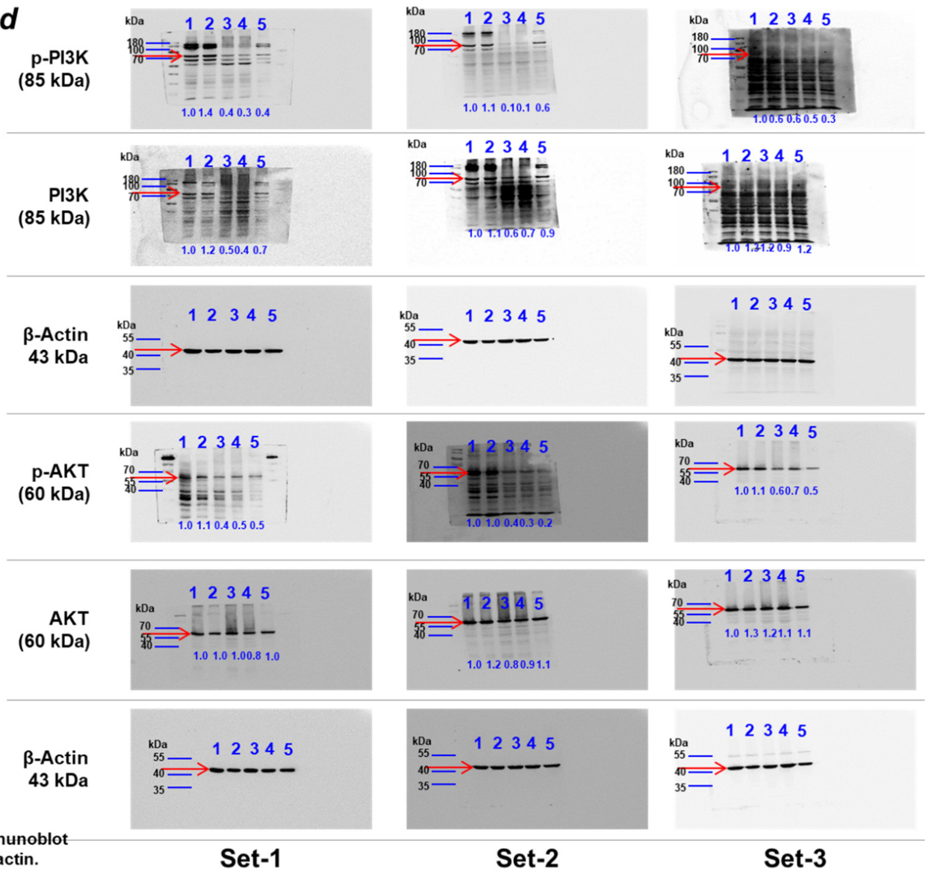

Each band intensity of immunoblot has been normalized to β-actin.

Fig 3 A continued

MIA PaCa-2

Lane 1: Mock
Lane 2: siNT
Lane 3: siHTR7
Lane 4: siTPH1
Lane 5: siEZH2

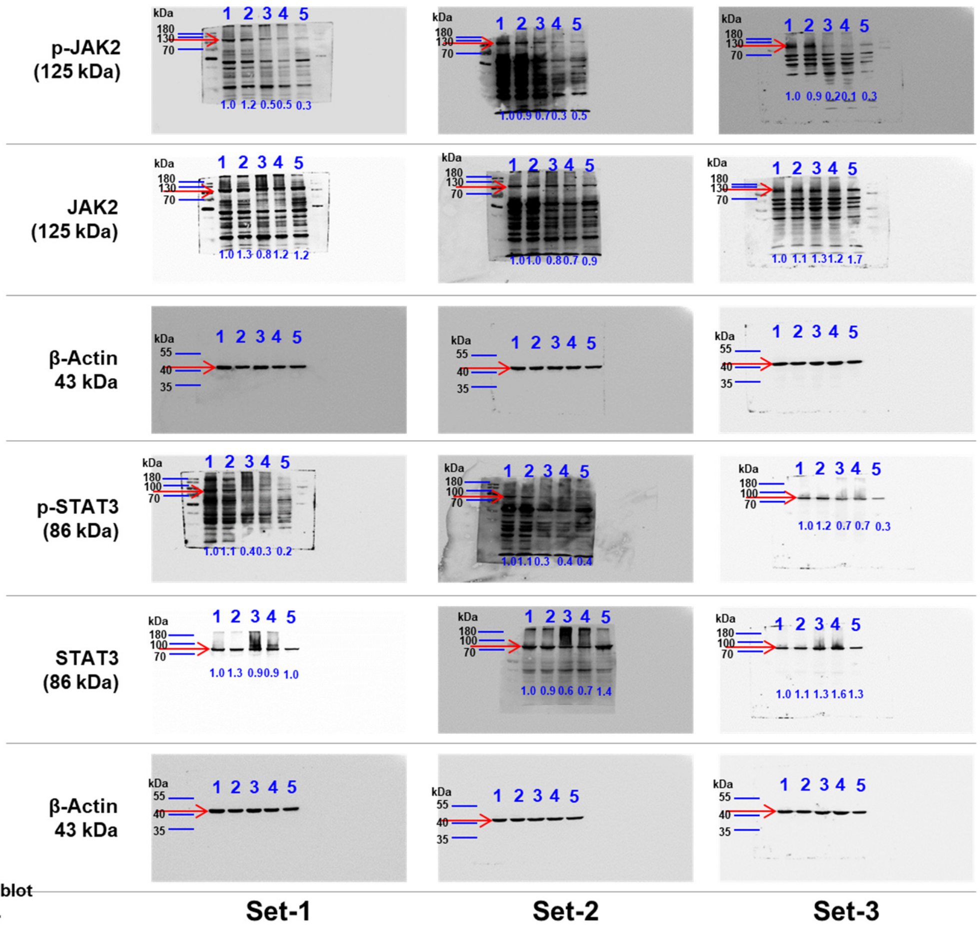

Each band intensity of immunoblot has been normalized to β-actin.

Fig 3 B

Lane 1: Vehicle
Lane 2: DZNep (5 µM)
Lane 3: DZNep (10 µM)
Lane 4: GSK126 (5 µM)
Lane 5: GSK126 (10 µM)

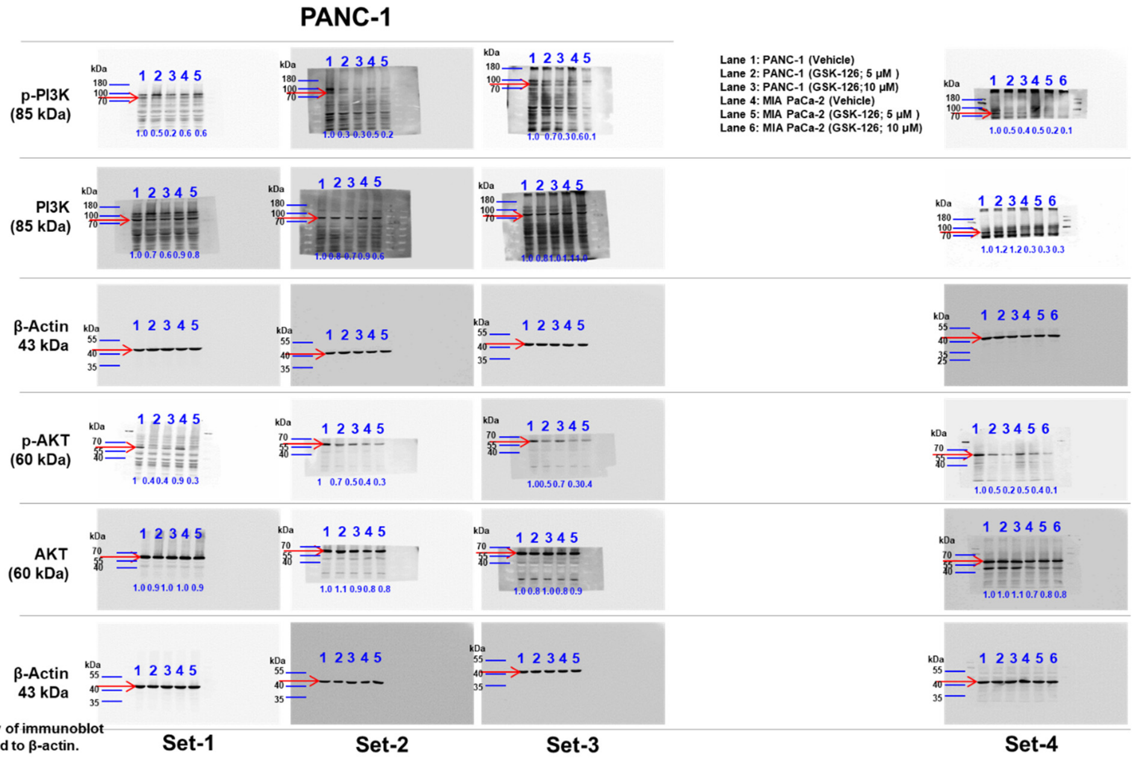

Each band intensity of immunoblot has been normalized to β-actin.

Fig 3 B Continued

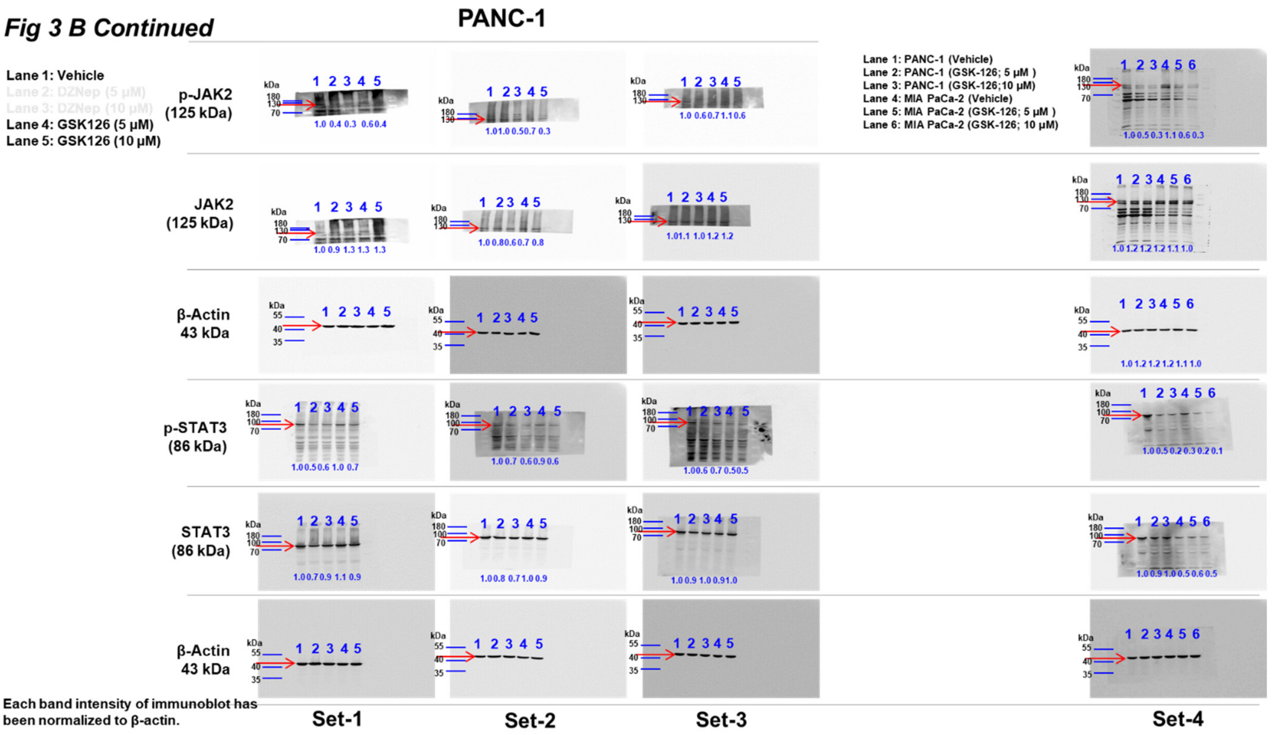

Fig 3 B Continued

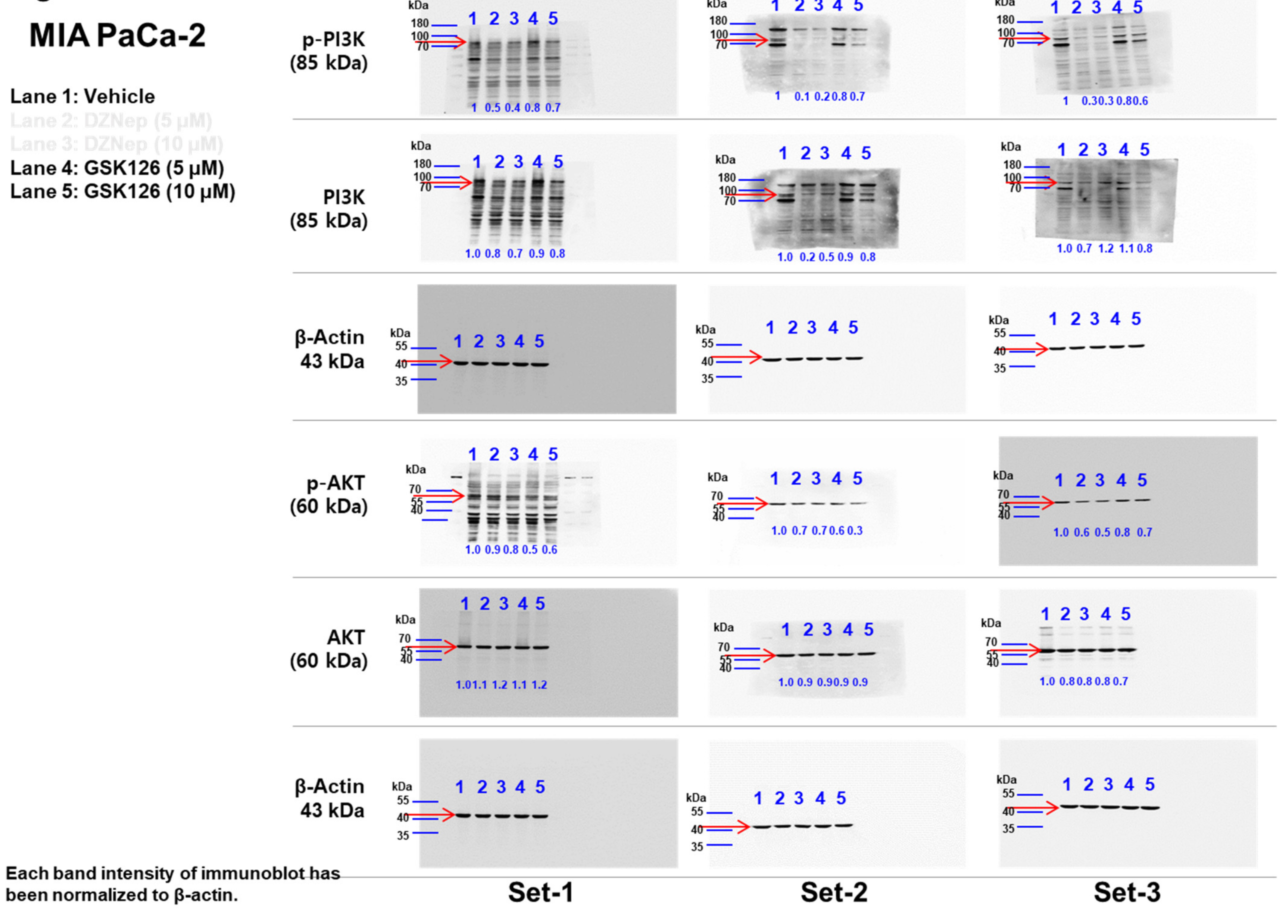

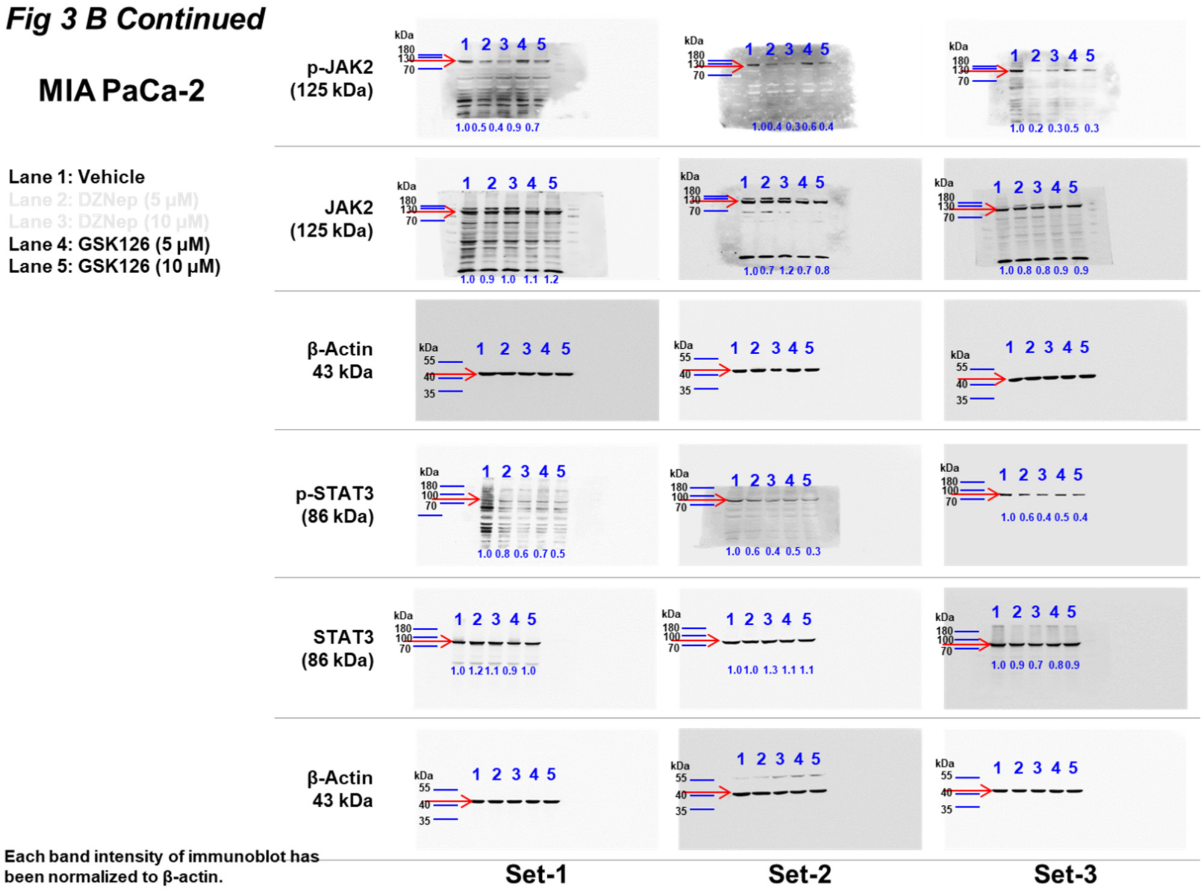

Figure S4. Uncropped Western Blot images for Figure 3.

Fig 4 C

PANC-1

Lane 1: Vehicle
Lane 2: Gemcitabine (1 µM)
Lane 3: Wortmannin (1 µM)
Lane 4: SC66 (1 µM)
Lane 5: Fedratinib (1 µM)
Lane 6: Stattic (1 µM)
Lane 7: SB-269970 (1 µM)
Lane 8: Telotristat (1 µM)
Lane 9: GSK-126 (1 µM)

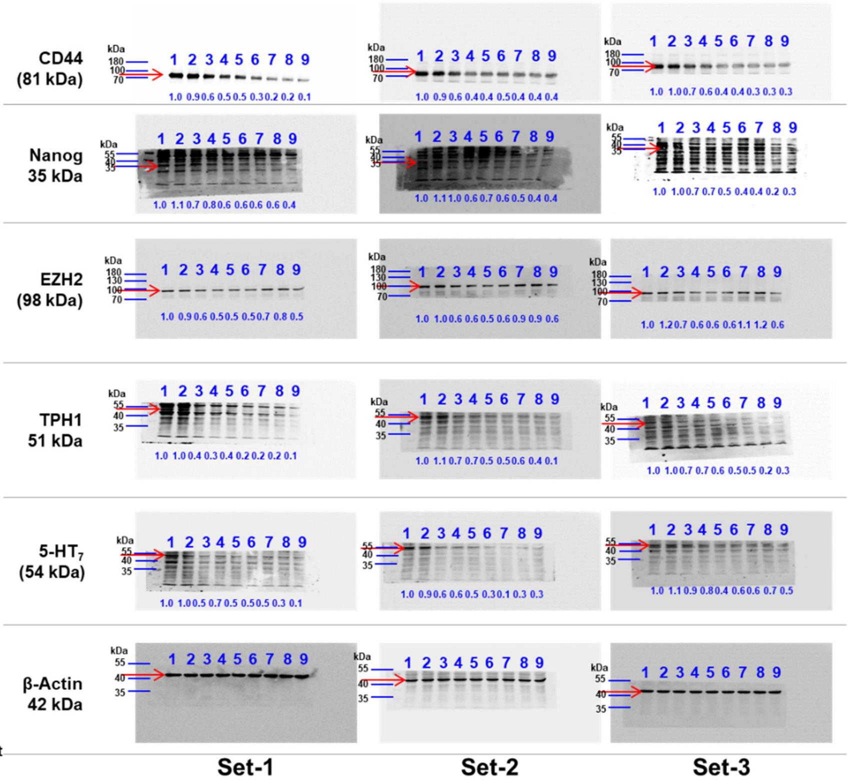

Each band intensity of immunoblot has been normalized to β-actin.

Fig 4 C continued

MIA PaCa-2

Lane 1: Vehicle
Lane 2: Gemcitabine (1 µM)
Lane 3: Wortmannin (1 µM)
Lane 4: SC66 (1 µM)
Lane 5: Fedratinib (1 µM)
Lane 6: Stattic (1 µM)
Lane 7: SB-269970 (1 µM)
Lane 8: Telotristat (1 µM)
Lane 9: GSK-126 (1 µM)

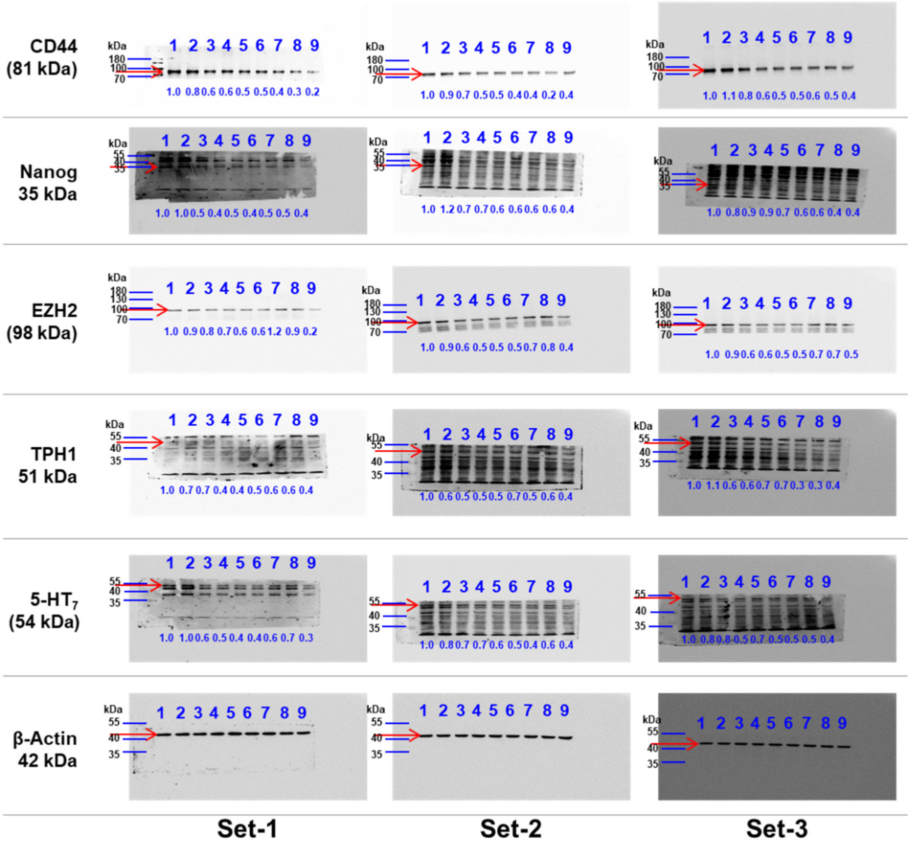

Each band intensity of immunoblot has been normalized to β-actin.

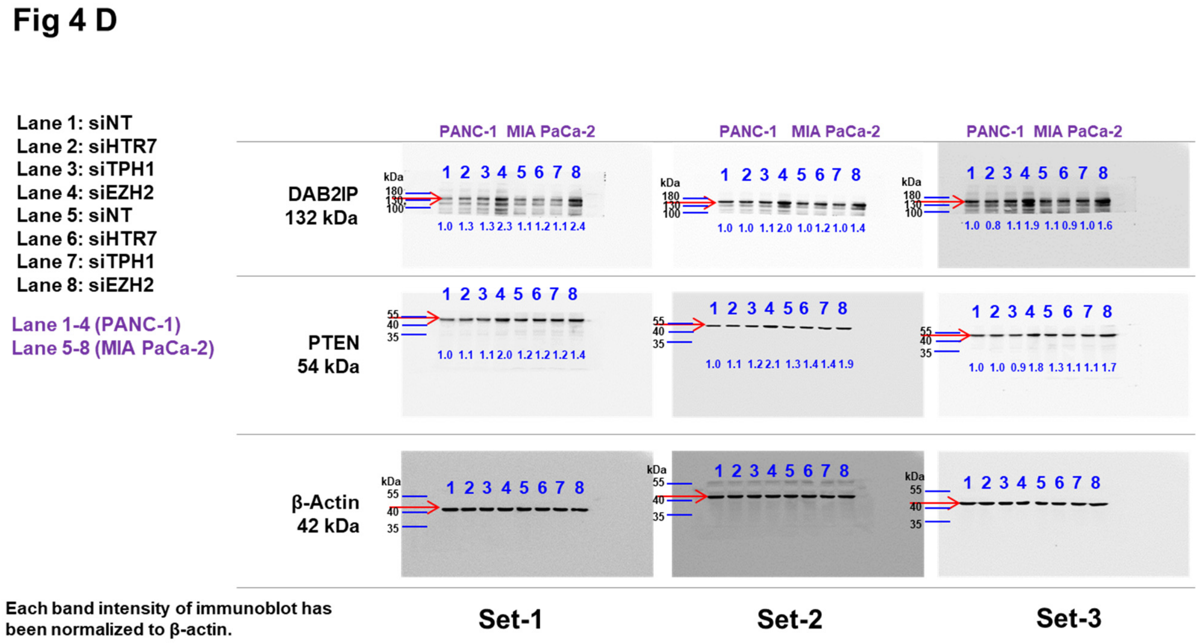

Figure S5. Uncropped Western Blot images for Figure 4.

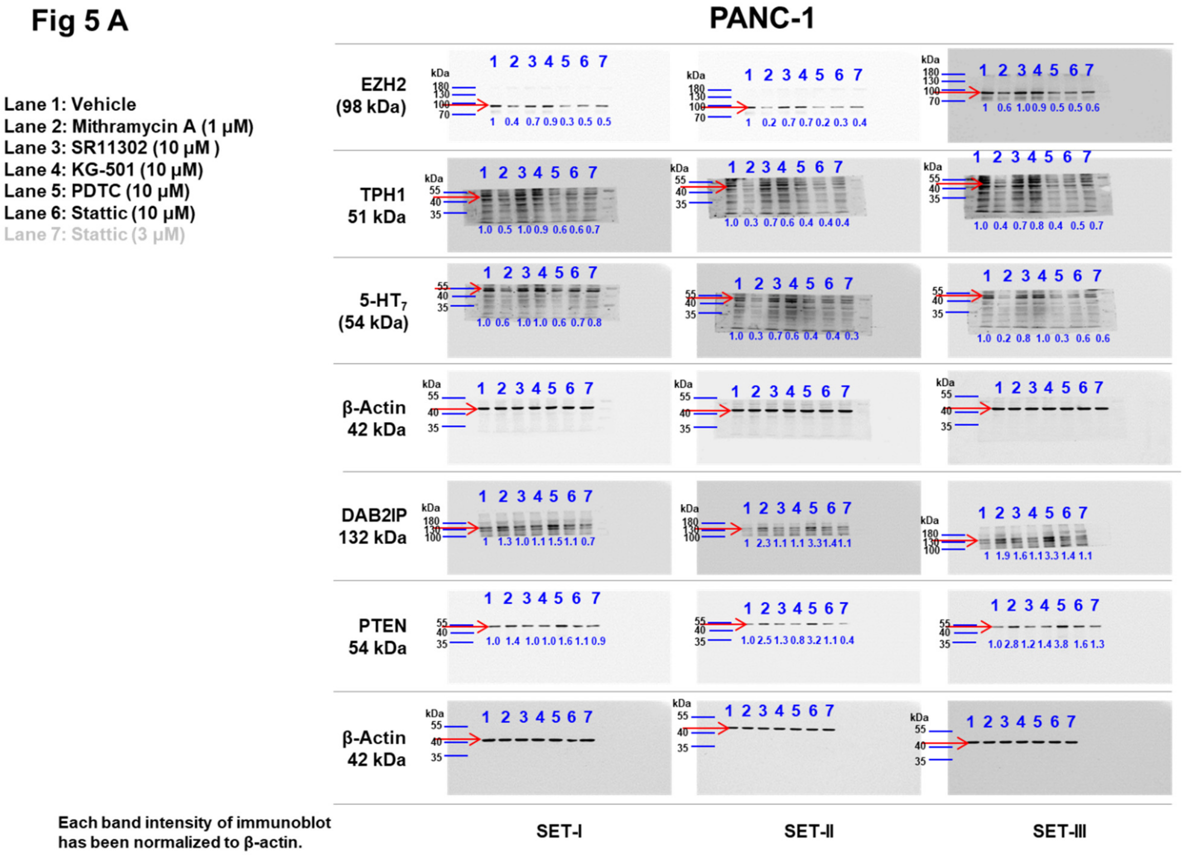

Fig 5 A Continued

Lane 1: Vehicle
Lane 2: Mithramycin A (1 μM)
Lane 3: SR11302 (10 μM)
Lane 4: KG-501 (10 μM)
Lane 5: PDTC (10 μM)
Lane 6: Stattic (10 μM)
Lane 7: Stattic (3 μM)

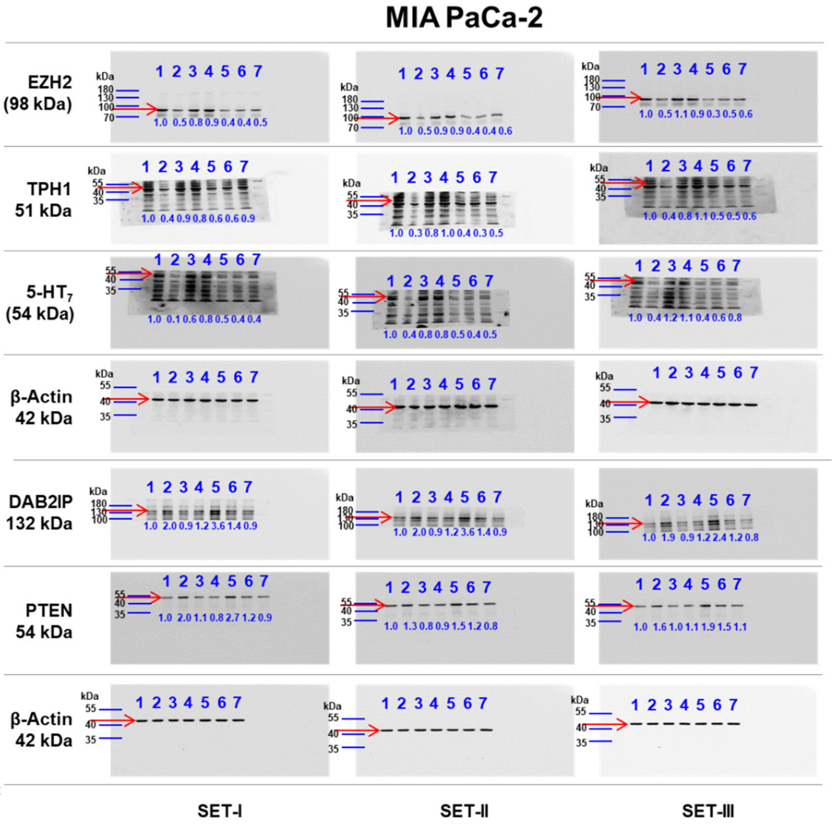

Each band intensity of immunoblot has been normalized to β-actin.

Fig 5 B

Lane 1: Vehicle
Lane 2: Gallein (10 μM)
Lane 3: Wortmannin (3 μM)
Lane 4: SC66 (3 μM)
Lane 5: Fedratinib (3 μM)
Lane 6: Stattic (3 μM)
Lane 7: SB-269970 (3 μM)
Lane 8: Telotristat (3 μM)
Lane 9: SB203580 (10 μM)

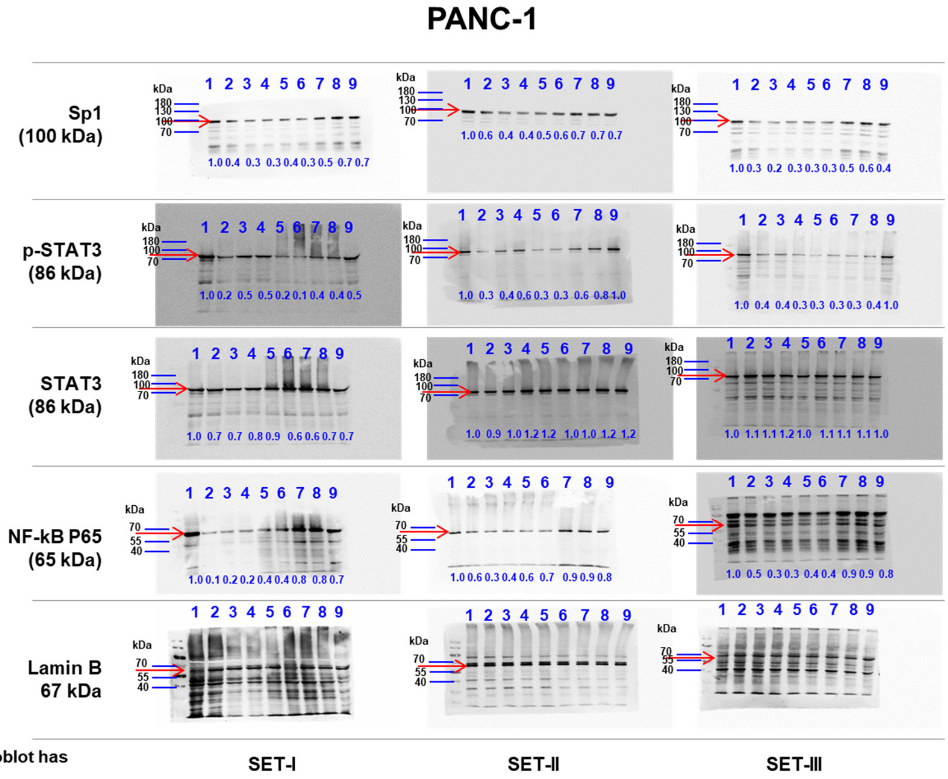

Each band intensity of immunoblot has been normalized to Lamin B.

Fig 5 B continued

Lane 1: Vehicle
Lane 2: Gallein (10 µM)
Lane 3: Wortmannin (3 µM)
Lane 4: SC66 (3 µM)
Lane 5: Fedratinib (3 µM)
Lane 6: Stattic (3 µM)
Lane 7: SB-269970 (3 µM)
Lane 8: Telotristat (3 µM)
Lane 9: SB203580 (10 µM)

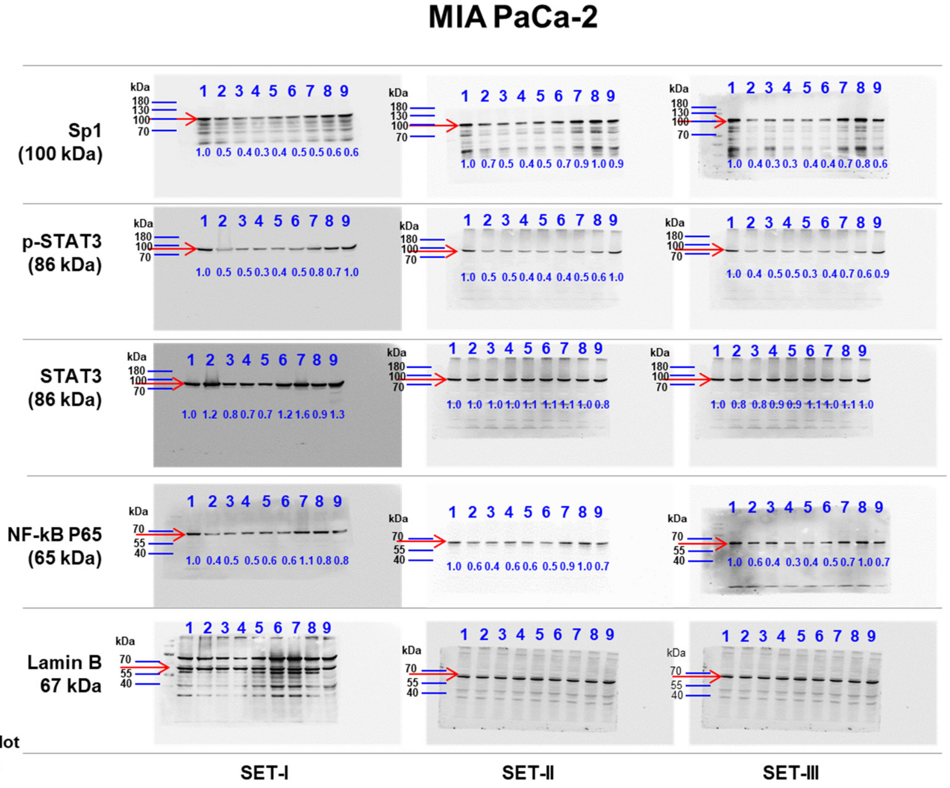

Each band intensity of immunoblot has been normalized to Lamin B.

Fig 5 C

Lane 1: Vehicle
Lane 2: 5-HT (10 µM)
Lane 3: 5-HT + DDA (100 µM)
Lane 4: 5-HT + Gallein (10 µM)
Lane 5: 5-HT + Wortmannin (3 µM)
Lane 6: 5-HT + SC66 (3 µM)
Lane 7: 5-HT + Fedratinib (3 µM)
Lane 8: 5-HT + Stattic (3 µM)
Lane 9: 5-HT + SB-269970 (3 µM)
Lane 10: 5-HT + Telotristat (3 µM)
Lane 11: 5-HT + SB203580 (10 µM)

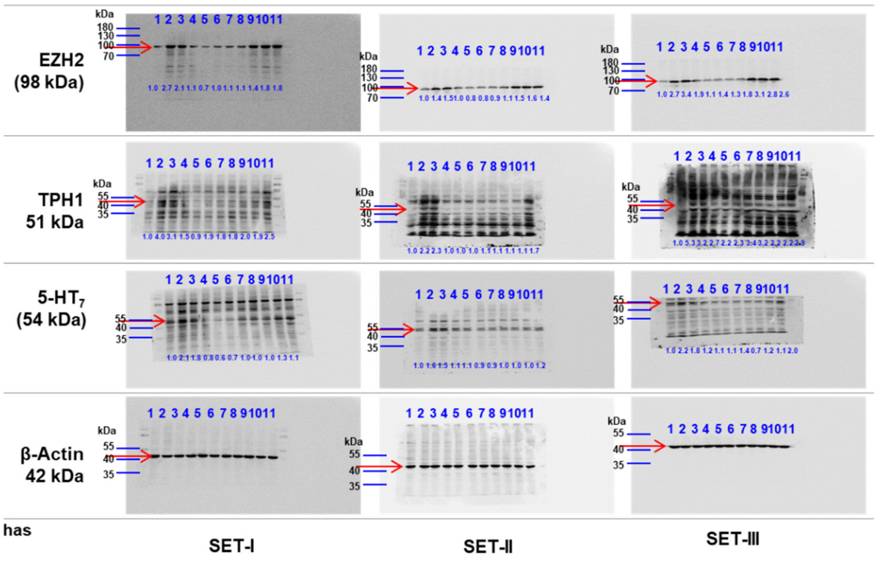

Each band intensity of immunoblot has been normalized to β-actin.

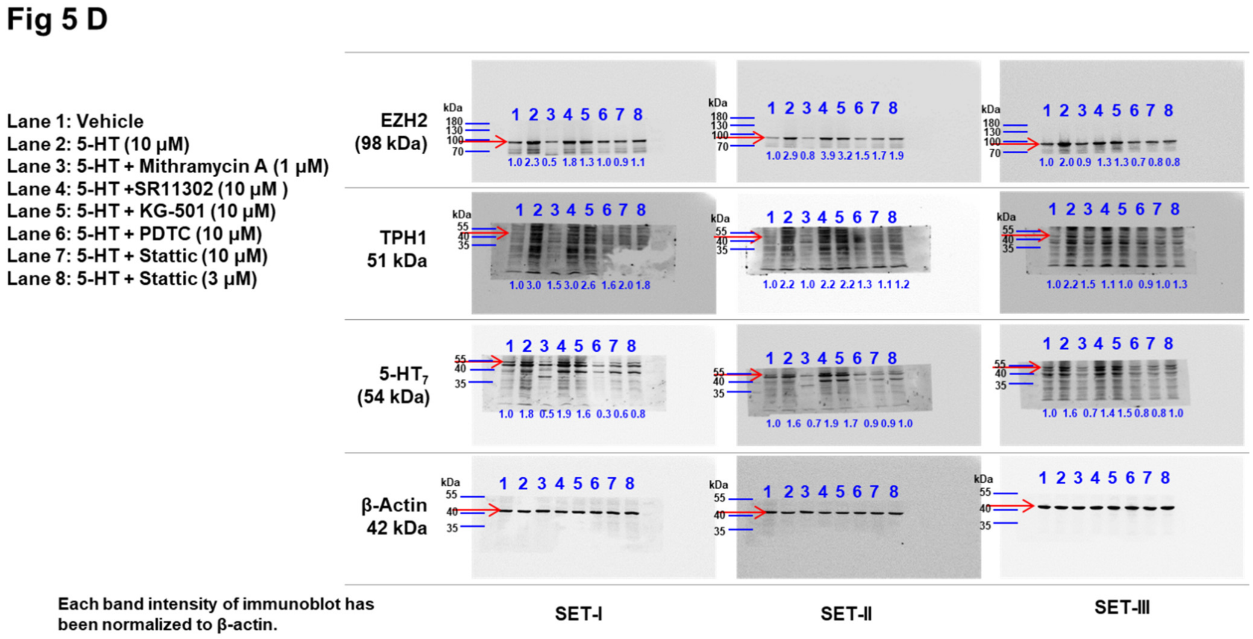

Figure S6. Uncropped Western Blot images for Figure 5.

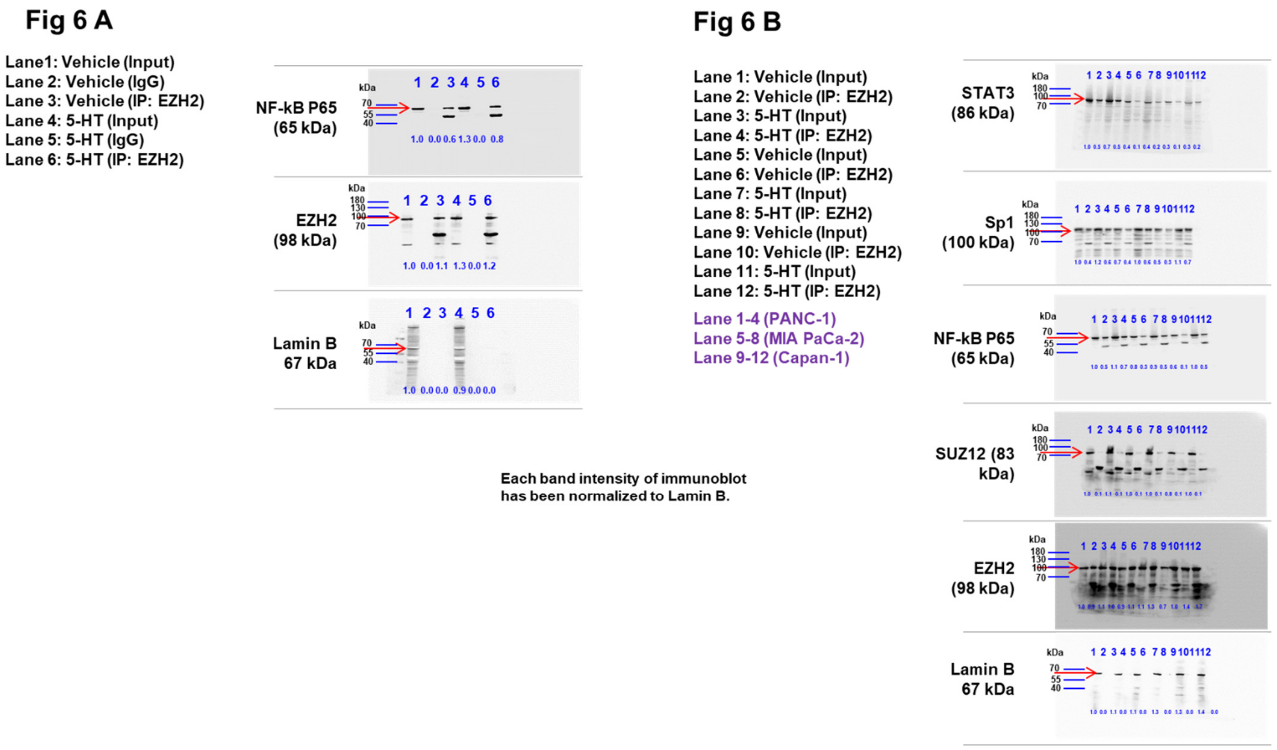

Fig 6 C

Lane1: Input
Lane 2: IgG
Lane 3: IP: Pan methyl lysine
Lane 4: Input
Lane 5: IgG
Lane 6: IP: Pan methyl lysine

Lane 1-3 (PANC-1)
Lane 4-6 (MIA PaCa-2)

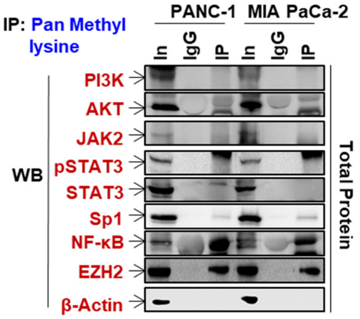

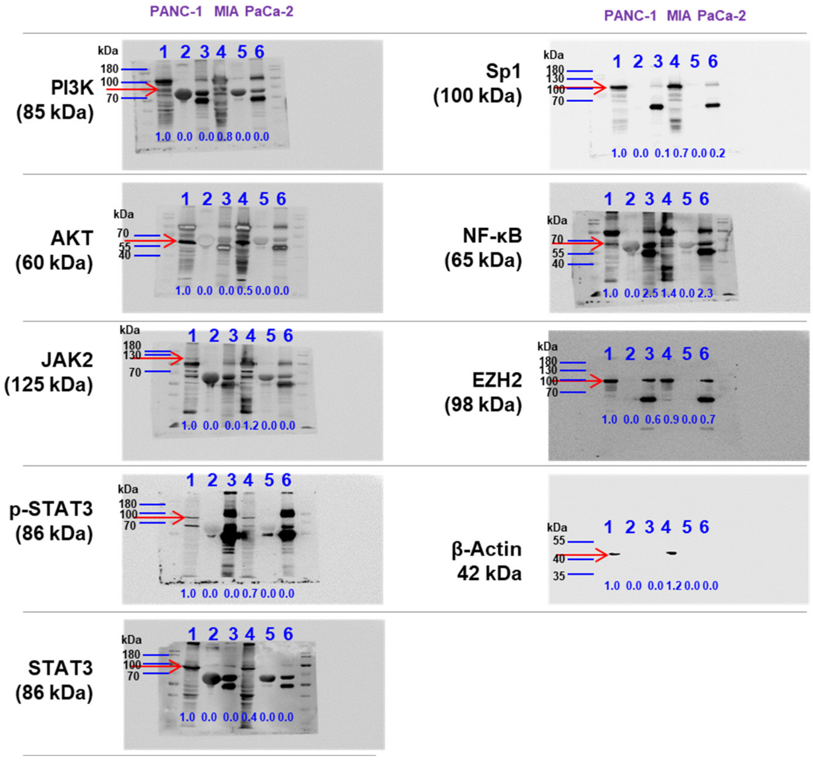

Fig 6 D

Lane1: Vehicle (Input)
Lane 2: Vehicle (IgG)
Lane 3: Vehicle (IP: Pan methyl lysine)
Lane 4: siEZH2 (Input)
Lane 5: siEZH2 (IP: Pan methyl lysine)
Lane 6: GSK-126 (Input)
Lane 7: GSK-126 (IP: Pan methyl lysine)
Lane 8: SB-269970 (Input)
Lane 9: SB-269970 (IP: Pan methyl lysine)
Lane 10: Telotristat (Input)
Lane 11. Telotristat (IP: Pan methyl lysine)

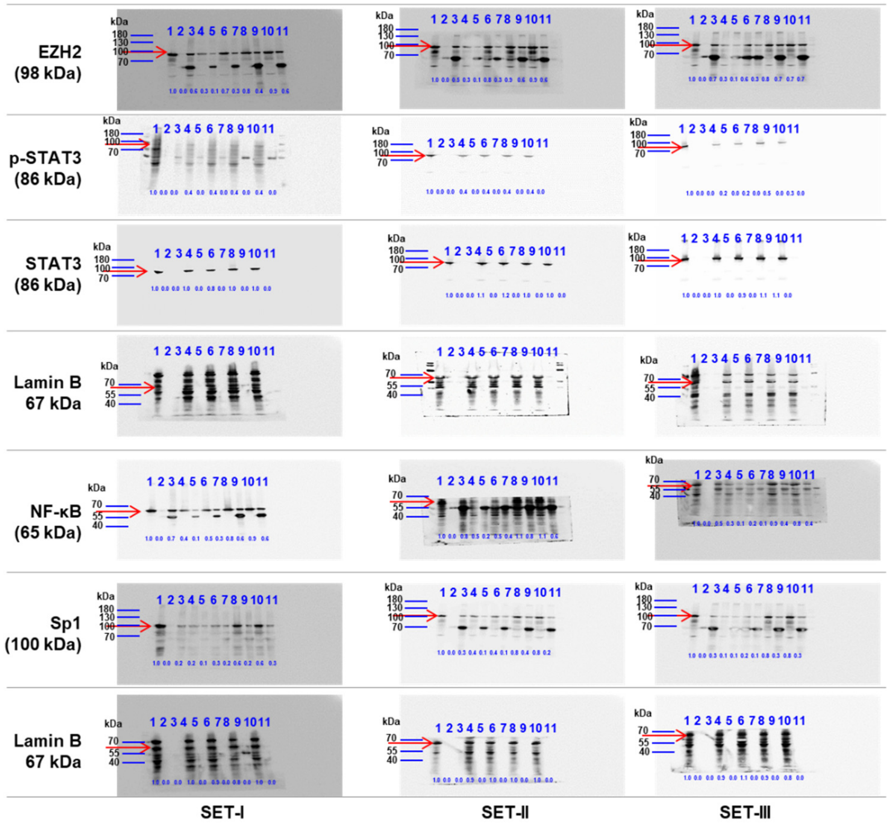

Figure S7. Uncropped Western Blot images for Figure 6.
